# Evaluation of monocyte distribution width as a predictive factor for early complications of pancreatic surgery (pancreaticoduodenectomy): a retrospective cohort study

**DOI:** 10.1186/s12893-025-03272-2

**Published:** 2025-11-03

**Authors:** Muhammet Berkay Sakaoglu, Tarkan Unek, Murat Ormen, Meltem Cetinkaya

**Affiliations:** 1 General Surgery Department, Dokuz Eylul University Medical Faculty Hospital, Balçova, İzmir Turkey; 2 Department of Biochemistry, Dokuz Eylul University Medical Faculty Hospital, Balçova, İzmir Turkey

**Keywords:** Monocyte distribution width, Early complications of pancreatic surgery, Anastomotic leak, Pancreatic fistula

## Abstract

**Background:**

The aim of this study was to evaluate the clinical utility of the monocyte distribution width (MDW) as an early diagnostic biomarker for detecting postoperative complications in pancreatic surgery patients. Complications from pancreatic surgery, particularly pancreatic fistulas, significantly reduce patient survival rates. Compared with conventional markers, changes in the MDW may be detected earlier, facilitating timely intervention and potentially improving patient outcomes.

**Methods:**

The MDW, C-reactive protein (CRP) level, and white blood cell (WBC) count were measured preoperatively and on postoperative days 1, 3, and 7. Complications—including clinically relevant pancreatic fistulas (CR-POPF) and anastomotic leaks—were classified using standardized criteria. Statistical analysis involved ROC curves and multivariate modelling to assess diagnostic accuracy and independent predictors. This retrospective analysis of a prospectively collected cohort included 82 patients who underwent elective pancreaticoduodenectomy (PD) for cancer at a single centre between May 2021 and March 2024.

**Results:**

In this cohort of 82 patients with prospective data collection and retrospective analysis who underwent PD, the MDW emerged as a significant early predictor of postoperative complications. On postoperative day 3, the MDW was independently associated with CR-POPF (AUC 0.781; OR 1.31, *p* = 0.044) and anastomotic leaks (ΔMDW days 0–3: OR 1.30, *p* = 0.015).

Compared with conventional markers, the MDW demonstrated superior diagnostic performance, with ROC AUC values ranging from 0.770 to 0.818 across different complications. A day 3 cut-off value of > 23.1 showed high sensitivity (84%) and yielded positive likelihood ratios of up to 3.7. Furthermore, the MDW on day 3 was moderately to strongly correlated with subsequent inflammatory markers, such as the CRP level, on day 7 (*r* = 0.468, *p* < 0.001). Multivariate models confirmed the independent prognostic value of the MDW for predicting overall complications, anastomotic leaks, and CR-POPF.

**Conclusion:**

Compared with the CRP level and WBC count, the MDW demonstrated superior and earlier predictive ability for detecting postoperative complications. Its elevation by day 3 provided early warning, especially for CR-POPF and leaks. As a rapid, cost-effective marker available from routine blood counts, the MDW may enhance postoperative monitoring and guide timely intervention.

**Supplementary Information:**

The online version contains supplementary material available at 10.1186/s12893-025-03272-2.

## Background

Currently, the only known curative treatment method for pancreatic cancer is surgical resection [1]. However, pancreatic surgery is a very complex and challenging process and therefore carries the risk of various complications. In the short term, these complications may lead to prolonged hospitalization, the need for interventional procedures, intensive care, or reoperation, and even sudden death. In the long term, the complications can delay the start of adjuvant chemotherapy. All these factors increase treatment costs and negatively affect patient survival. Among the most feared complications in pancreatic surgery are pancreatic fistulas and anastomotic leaks. Owing to their life-threatening nature, pancreatic fistulas have been extensively investigated in numerous scientific studies [2, 3].

Early detection or prediction of complications after pancreatic surgery may allow measures to be taken to reduce downstream effects. In this context, many studies have been performed and important steps have been taken towards the identification of standardized risk factors. Furthermore, various algorithms have been developed to facilitate the diagnosis and treatment of complications [4].

Therefore, early detection of complications and knowledge of their prognosis are important.

Monocytes play a pivotal role in systemic inflammatory responses [5–7]. MDW, a novel biomarker measured via volume, conductivity, and scatter (VCS) technology, can be used to quantify changes in monocyte volume heterogeneity with high precision. Its integration into routine complete blood counts—without requiring additional blood sampling—and Food and Drug Administration (FDA) approval for sepsis diagnosis [8–11] make MDW an attractive candidate for postoperative monitoring. This study focuses on MDW for two key reasons. First, modern VCS technology now allows for highly precise and cost-effective measurement of subtle monocyte volume changes through MDW measurements. Second, these cellular alterations can serve as early indicators of postoperative complications, often days before they become apparent in routine blood tests or clinical symptoms. The exceptional sensitivity of MDW could provide a critical diagnostic window, potentially enabling preemptive clinical intervention and offering a significant advantage in postoperative patient management. In addition, MDW measurement can prevent unnecessary examinations by identifying patients with no complications and who follow a normal course of recovery. Given all of these factors, we plan to introduce MDW as a new parameter in the surgical literature with the aim of contributing to patient survival and reducing costs.

We found only one surgical study in the literature that examined MDW, which focused on distinguishing between simple and complicated diverticulitis [12]. As no previous research has assessed MDW in the context of pancreatic surgery, our study represents the first attempt to investigate its role in predicting postoperative complications in this setting.

## Methods

### Patient data and sample collection

This retrospective analysis of a prospectively collected cohort analysed all consecutive patients (*n* = 82) who underwent PD for oncological indications at Dokuz Eylül University Hospital’s Department of General Surgery between May 1, 2021, and March 1, 2024. Patients who underwent emergency procedures were excluded from the study. Prior to inclusion, written informed consent was obtained from all participants.

The study period commencement date was selected to coincide with the implementation of routine MDW measurement capability in our clinical laboratory. The study population comprised only elective, cancer-related PD cases, ensuring homogeneity in the surgical indications.

To minimize bias, laboratory personnel performing MDW measurements were blinded to the clinical outcomes, whereas clinicians assessing postoperative complications were blinded to the MDW measurement results.

*Blinding*: Although MDW values are automatically generated by the hematology analyzer, they are not included in routine complete blood count reports, and clinicians were therefore not exposed to MDW values at any time. Laboratory staff extracted MDW values under anonymized identifiers and were blinded to clinical outcomes. This approach ensured independent assessment of exposure and outcomes, providing robust blinding suitable for a retrospective cohort study.

Comprehensive patient data were extracted through a systematic review of electronic medical records and paper-based hospital archives. All the collected data were anonymized and processed in strict compliance with Turkey’s Personal Data Protection Law (KVKK) No. 6698 and institutional data governance policies.

The study protocol received ethical approval from Dokuz Eylül University Faculty of Medicine’s Non-Interventional Clinical Research Ethics Committee (Approval No: 2024/05–19; Date: February 7, 2024). Perioperative data were prospectively collected with patient consent but the data analysis was performed retrospectively after complete clinical follow-up.

Complications, pancreatic fistulas and anastomotic leaks were identified as independent variables. The MDW, CRP, and WBC measurement results at preoperative day 0 and postoperative days 1, 3, and 7 were documented. Blood samples for MDW, CRP, and WBC were collected at standardized times (preoperatively and at 6–8 a.m. on postoperative days 1, 3, and 7) to minimize diurnal variation and ensure comparability across patients.

Complications occurring within 1-month post-surgery were included as early complications, as stated in the literature.

The Clavien‒Dindo (CD) classification was used for complications. CD I and CD II were categorized into one group, and CD III, CD IV and CDV were categorized into another group. Early mortality was defined as death within the first month after surgery. Thus, CD V was evaluated separately as early mortality. For the classification of pancreatic fistulas, the International Study Group of Pancreatic Surgery (ISGPS) definitions for postoperative pancreatic fistula (POPF) classification were used. CR-POPF were classified as POPF B or POPF C (8,9). POPF was defined by the combination of a drain amylase level measured postoperatively that was at least three times higher than the serum amylase level and clinical deterioration [13, 14]. The definition and classification of biliary fistula by the International Study Group for the Surgery of the Liver (ISGLS) was used for hepaticojejunostomy leaks [15]. Complications, anastomotic leaks and CR-POPF were evaluated as dependent variables in the study. Since the most common period for complications was the first 7 days after surgery, we analysed the relationships among complications, CR-POPF and anastomotic leaks, the MDW, CRP, and WBC results on preoperative day 0 and postoperative days 1, 3 and 7 and the changes in these parameters between days. All complications were evaluated. Anastomotic leaks included CR-POPF, hepaticojejunostomy leaks, gastrojejunostomy leaks and jejunojejunostomy leaks. Patients with lymphatic leaks were included not in the anastomotic leak group but in the complication group. Only CR-POPF were included in the pancreatic leak group.

The Unicel DxH 800 Coulter Cellular Analysis System, a haematology analyser manufactured by Beckman Coulter, Inc.©, was used for the MDW measurements. All hematological analyses were performed in our accredited laboratory with regular internal and external quality control procedures for the hematology analyzer to ensure reliability of MDW values. AI tools were used only for language and grammar editing.

### Operative technique

All operations were performed by the same surgical team. Wirsungojejunostomy anastomosis was performed as an end-to-side Wirsungojejunostomy between the jejunum and pancreatic tissue by the classic technique with 8 5 − 0 polyglyconate (PG) sutures after 4 − 0 polydioxanone (PDS) background sutures were placed in the jejunum. End-to-side single-layer hepaticojejunostomy was performed with 5 − 0 PDS sutures approximately 5 cm distal to the wirsungojejunostomy. An antecolic, double-layer gastrojejunostomy was performed 50 cm distal to the hepaticojejunostomy with 3 − 0 PG, and a double-layer, side-to-side jejunojejunostomy (Braun anastomosis) was performed 15 cm distal to the gastrojejunostomy with 3 − 0 PG. No stapler was used in any of these anastomoses. Drains were placed in the fossa subhepatica dextra and anterior aspect of the gastrojejunostomy.

All procedures were performed by a high-volume hepatopancreatobiliary surgical team, each surgeon having more than 10 years of experience, with the unit performing > 50 pancreatic resections annually. Drain removal was considered when the output was < 50 mL/day, serous in nature, and with drain amylase < 3× serum amylase. Patients were monitored with daily clinical examination and routine laboratory testing during the first postoperative week.

### Statistical analysis

Statistical analyses were performed using SPSS Statistics version 29.0 (IBM Corp., Armonk, NY, USA), PAST version 4.03 (Hammer Ø, Harper D.A.T., Ryan P.D., 2023. *Paleontological Statistics*, University of Oslo, Norway), and MedCalc version 22 (Acacialaan 22, B-8400 Ostend, Belgium).

The normality of univariate data distributions was assessed by the Shapiro‒Wilk‒Francia test, and the homogeneity of variances was evaluated via Levene’s test. For multivariate data, Mardia’s test (Dornik and Hansen omnibus) was applied to test for multivariate normality, whereas Box’s M test was used to assess the equality of covariance matrices.

For dependent quantitative variables with more than two repeated measurements, Friedman’s test (with Monte Carlo simulation) and repeated-measures ANOVA were used for comparisons. In cases with two repeated measurements, the Wilcoxon signed-rank test (with Monte Carlo simulation) and the paired samples t test (with bootstrapping) were employed, depending on the data distribution.

Variables found to be significantly associated with clinical outcomes—including overall complication status, anastomotic leak, POPF, and surgical site infection (SSI)—were selected using the best subset selection method. The diagnostic accuracy of these selected variables was assessed using receiver operating characteristic (ROC) curve analysis. The sensitivity, specificity, positive predictive value (PPV), and negative predictive value (NPV) were calculated on the basis of optimal cut-off values for each clinical outcome.

To explore potential causal relationships and predict clinical outcomes, multiple machine learning models were developed and compared. Eight machine learning classifiers were implemented (logistic regression, decision tree, random forest, support vector machine (SVM) with RBF kernel, k-nearest neighbors, naïve Bayes, linear discriminant analysis (LDA), and quadratic discriminant analysis (QDA)). All models were evaluated using stratified 5-fold cross-validation. Model performance was assessed using accuracy, sensitivity, specificity, precision, F1-score, and ROC-AUC.

Continuous variables are presented as the mean ± standard deviation or median (minimum–maximum), as appropriate. Categorical variables are expressed as frequencies and percentages (n, %). All the statistical tests were two-sided, and a *p* value < 0.05 was considered indicative of statistical significance.

A post-hoc power analysis was performed to assess the adequacy of sample size.

To control for Type I error across multiple comparisons, the Benjamini–Hochberg false discovery rate (FDR) correction was applied within each outcome family. Bonferroni correction was not used, as it is considered overly conservative and may substantially increase the risk of Type II error in small to moderate cohorts.

## Results

This study included 82 patients. The median age of the patients was 64 years (27–84); 49 (59.8%) of the patients were male, and 33 (40%) were female. The median follow-up period was 11 (0.5–34) months, and no patients were lost to follow-up within the first 30 days. The 1- and 2-year survival rates were 70% and 61%, respectively. In supplementary survival analyses, patients who developed early complications had significantly poorer overall survival compared with those without complications (log-rank *p* = 0.003; Supplementary Fig. 1).

Twenty-five (30.5%) patients had no comorbidities. Twenty-nine (35.4%) patients had diabetes mellitus, 15 (18.3%) patients had hypertension, 6 (7.3%) patients had coronary artery disease, 2 (2.4%) patients had pulmonary disease, and 2 (2.4%) patients had chronic kidney disease.

Forty-two (51.2%) of the patients were smokers.

Forty-nine (58.8%) patients had preoperative bile drainage. Twenty-nine (35.3%) patients underwent bile drainage by endoscopic retrograde cholangiopancreatography (ERCP) with stent placement, and 20 (24.3%) patients underwent bile drainage with a percutaneous transhepatic biliary drainage catheter.

The pancreatic tissue characteristics were as follows: 8 (9.7%) patients had hard, 47 (57.3%) patients had medium, and 27 (32.9%) patients had soft pancreatic tissue. The mean pancreatic main duct diameter was 4 mm (Table 1).

**Table 1:** Comparison of demographic and clinical features by complications, anastomotic leaks, and pancreatic fistulas

Variables	Complication, *n* (%)		*P* value	Anastomotic leak, *n* (%)		*P* value	Pancreatic fistula, *n* (%)		*P* value
	Absent	Present		Absent	Present)		No leak and biochemical leak	POPF B ve C	
Age	59 (27–82)	66 (43–84)	0.063^a^	63 (27–82)	68.5 (43–84)	0.137^a^	63.5 (27–82)	71.5 (43–84)	0.142^a^
Sex			0.172^b^			0.999^b^			0.302^b^
Female	16 (50)	17 (34)		27 (40.9)	6 (37.5)		27 (37.5)	6 (60)	
Male	16 (50)	33 (66)		39 (59.1)	10 (62.5)		45 (62.5)	4 (40)	
Smoking			0.651^b^			0.583^b^			**0.046** ^c^
Absent	17 (53.1)	23 (46)		31 (47)	9 (56.3)		32 (44.4)	8 (80)	
Present	15 (46.9)	27 (54)		35 (53)	7 (43.8)		40 (55.6)	2 (20)	
Comorbidity			0.999^b^			0.999^c^			0.716^c^
Absent	10 (31.3)	15 (30)		20 (30.3)	5 (31.3)		23 (31.9)	2 (20)	
Present	22 (68.8)	35 (70)		46 (69.7)	11 (68.8)		49 (68.1)	8 (80)	
Clavien‒Dindo			**< 0.001**			**< 0.001**			**< 0.001**
1–2	32 (100)	21 (42)		52 (78.8)	1 (6.3)		52 (72.2)	1 (10)	
3–4−5	0 (0)	29 (58)		14 (21.2)	15 (93.8)		20 (27.8)	9 (90)	
Early mortality			0.277^c^			0.096^c^			**0.038** ^c^
Absent	32 (100)	47 (94)		65 (98.5)	14 (87.5)		71 (98.6)	8 (80)	
Present	0 (0)	3 (6)		1 (1.5)	2 (12.5)		1 (1.4)	2 (20)	
Pancreatic texture			**0.047** ^d^			0.101^d^			**0.038** ^d^
Soft	12 (37.5)	15 (30)		18 (27.3)	9 (56.3)		20 (27.8)	7 (70)	
Intermediate	14 (43.8)	33 (66)		41 (62.1)	6 (37.5)		44 (61.1)	3 (30)	
Hard	6 (18.8)	2 (4)		7 (10.6)	1 (6.3)		8 (11.1)	0 (0)	
Preoperative biliary drainage			**0.002** ^d^			0.728^d^			0.574^d^
Absent	20 (62.5)	13 (26)		25 (37.9)	8 (50)		28 (38.9)	5 (50)	
ERCP stent	5 (15.6)	24 (48)		24 (36.4)	5 (31.3)		27 (37.5)	2 (20)	
PBD	7 (21.9)	13 (26)		17 (25.8)	3 (18.8)		17 (23.6)	3 (30)	
Blood transfusion			0.633^b^			0.999^b^			0.718^c^
Absent	23 (71.9)	33 (66)		45 (68.2)	11 (68.8)		50 (69.4)	6 (60)	
Present	9 (28.1)	17 (34)		21 (31.8)	5 (31.3)		22 (30.6)	4 (40)	
Number of RBC transfusions^*^	0 (0–4)	1 (0–9)	**0.010** ^a^	1 (0–5)	2 (0–9)	**0.004** ^a^	1 (0–7)	3 (0–9)	**0.008** ^a^
Number of FFP transfusions^*^	0 (0–8)	2 (0–23)	**0.027** ^a^	1 (0–11)	4 (0–23)	**0.002** ^a^	1 (0–11)	4.5 (0–23)	**0.026** ^a^
Pre-op albumin	3.7 (3.01–5.1)	3.5 (1.8–4.5)	**0.029** ^a^	3.6 (1.8–5.1)	3.45 (2.73–4.5)	0.530^a^	3.6 (1.8–5.1)	3.15 (2.73–4.5)	0.257^a^
Intraoperative blood loss, mL	225 (50–650)	250 (100–650)	0.518^a^	250 (50–650)	200 (150–650)	0.910^a^	250 (50–650)	225 (150–650)	0.638^a^
Pancreatic duct diameter, mm	3 (2–15)	4 (2–9)	0.373^a^	4 (2–15)	3 (2–8)	**0.002** ^a^	4 (2–15)	3 (2–3)	**< 0.001** ^a^
Mortality			**0.022** ^b^			**0.001** ^c^			0.134^c^
Alive	28 (87.5)	31 (62)		53 (80.3)	6 (37.5)		54 (75)	5 (50)	
Exitus	4 (12.5)	19 (38)		13 (19.7)	10 (62.5)		18 (25)	5 (50)	
Length of hospital stay	11 (7–25)	20 (9–53)	**< 0.001** ^a^	13 (7–43)	26.5 (12–53)	**< 0.001** ^a^	13.5 (7–43)	25 (16–53)	**< 0.001** ^a^
Follow-up (Months)	13.5 (1–35)	8 (1–34)	**0.002** ^a^	11 (1–35)	8 (1–29)	0.120^a^	11 (1–35)	5.5 (1–29)	0.212^a^

*Abbreviations*: *ERCP* Endoscopic retrograde cholangiopancreatography, *FFP* Fresh frozen plasma, *PBD* Percutaneous biliary drainage, *POPF* Postoperative pancreatic fistula, *RBC* Red blood cell

Notes:

^a^ Mann‒Whitney U test

^b^ Chi-square test

^c^ Fisher’s exact test

^d^ Friedman fisher test

Statistically significant *p* values (< 0.05) are highlighted in bold for clarity

One (1.2%) patient underwent PD for a neuroendocrine tumour, and 2 metastasectomies were performed in the liver, with a diameter of approximately 1 cm. No additional organ resection was performed in the other patients.

Three (3.6%) patients had a history of vascular resection.

Fifty-three (64.6%) patients had CD I and CD II complications. Another 29 (35.4%) patients had CD III, CD IV, and CDV complications. Three (3.6%) patients had CD V complications and experienced early mortality (Table 1). The causes of death in these patients were POPF C in two patients and sepsis in the other patient.

There were 59 complications in 50 (61%) of the patients. Thirty-two patients had no complications. Superficial SSI was observed in 26 (31.7%) patients, and deep SSI was observed in 7 (8.5%) patients.

Pancreatic leakage was observed in 21 (25.6%) patients. Among these leaks, 11 (13.4%) were biochemical leaks, 7 (8.5%) were POPF type B leaks, and 3 (3.7%) were POPF type C leaks.

No patient had a type C hepaticojejunostomy leak. Six (7%) patients had a type B hepaticojejunostomy leak.

Four (4.8%) patients had lymphatic leaks.

Three (3.6%) patients exhibited positive blood cultures and sepsis.

Atelectasis and pneumonia developed in 2 (2.4%) patients with preoperative lung disease.

One (1.2%) patient experienced upper gastrointestinal bleeding (Table 2).

**Table 2:** Types and distribution of postoperative complications

Type of complication	N (%)
Superficial surgical site infection	26 (31.7)
Deep surgical site infection	7 (8.5)
Postoperative pancreatic fistula (POPF B)	7 (8.5)
Postoperative pancreatic fistula (POPF C)	3 (3.7)
Hepaticojejunostomy leakage (Type B)	6 (7)
Lymphatic leakage	4 (4.8)
Sepsis	3 (3.6)
Pneumonia	2 (2.4)
Upper gastrointestinal bleeding	1 (1.2)

*Abbreviations*: *n* number, *POPF* Postoperative pancreatic fistula

### Univariate analysis results

Compared with the CRP level and WBC count, the MDW demonstrated superior predictive performance across multiple postoperative outcomes. For general complications, the MDW was statistically significant at all measured time points (postoperative days 0, 1, 3, and 7), whereas the CRP level reached significance only on days 0, 3, and 7, and the WBC count changed only between days 1 and 7.

In detecting anastomotic leaks, the MDW again outperformed other biomarkers, showing significance at days 3 and 7 and dynamic changes between days 0–3, 0–7, and 1–3. In contrast, the CRP level and WBC count were significant mainly on day 7 and in fewer interval comparisons.

For CR-POPF, the MDW exhibited broader diagnostic relevance, with significant values observed on days 3 and 7 and across multiple change intervals (0–3, 1–3, 1–7). The CRP level and WBC count showed more limited significance, both in terms of timing and predictive consistency (Table 3).

**Table 3:** Perioperative MDW, CRP, and WBC dynamics and association with complications, anastomotic leaks, and fistulas

Variables	Complication		*P* value	Anastomotic leak		*P* value	Pancreatic fistula		*P* value
	Absent	Present		Absent	Present		No leak and biochemical leak	POPF B ve C	
MDW									
Day 0	19.4 (15.7–26.9)	20.8 (15.5–27.7)	**0.007** ^a^	20.3 (15.7–27.7)	20 (15.5–24.7)	0.590^a^	20.3 (15.5–27.7)	20.9 (17.3–24.7)	0.497^a^
Day 1	20.7 (16.2–27.7)	21.9 (17–36)	**0.008** ^a^	20.9 (16.2–31.5)	22.2 (18.9–36)	0.131^a^	21.2 (16.2–31.5)	21.4 (18.9–36)	0.928^a^
Day 3	22.8 (19.2–31.2)	25.8 (21.2–32.3)	**< 0.001** ^a^	23.4 (19.2–31.2)	28 (22.4–32.3)	**0.001** ^a^	23.5 (19.2–31.2)	28.6 (22.4–32.3)	**0.003** ^a^
Day 7	21.4 (16.4–25.4)	23.9 (19.1–37.3)	**< 0.001** ^a^	22.8 (16.4–37.3)	24.9 (21.5–35)	**0.004** ^a^	22.9 (16.4–37.3)	24.9 (22.1–35)	**0.007** ^a^
MDW change									
Day (0–1)	1.5 (− 3.8–9)	1.6 (− 8–13.9)	0.879^a^	1.3 (− 8–9)	2 (− 3.8–13.9)	0.262^a^	1.6 (− 8–13.9)	1.4 (− 3.8–12.6)	0.619^a^
Day (0–3)	3.5 (− 4–13.2)	4.3 (− 3.1–14.7)	0.285^a^	3.5 (− 4–13.2)	7.9 (− 1–14.7)	**0.001** ^a^	3.9 (− 4–14.7)	7.9 (− 1–12.3)	**0.024** ^a^
Day (0–7)	2 (− 5.8–6.7)	2.6 (− 3.9–12.6)	0.142^a^	2 (− 5.8–12.6)	5 (− 0.3–12.3)	**0.008** ^a^	2.5 (− 5.8–12.6)	4.8 (− 0.3–12.3)	0.168^a^
Day (1–3)	2.2 (− 2.7–10.7)	3.6 (− 13.6–11.7)	0.145^a^	2.8 (− 7.8–10.7)	6.3 (− 13.6–11.7)	**0.047** ^a^	2.8 (− 7.8–10.7)	6.9 (− 13.6–11.7)	**0.003** ^a^
Day (1–7)	1.1 (− 4.3–5)	2.6 (− 12.9–16.1)	0.090^a^	1.2 (− 7.6–14.3)	3.2 (− 12.9–16.1)	0.213^a^	1.1 (− 7.8–14.3)	3.4 (− 12.9–16.1)	**0.012** ^a^
Day (3–7)	−0.6 (− 8.4–3)	−1.6 (− 8.9–12.1)	0.924^a^	−0.8 (− 8.9–12.1)	−1.9 (− 7.4–5.3)	0.404^a^	−0.9 (− 8.9–12.1)	−1.9 (− 7.4–4.4)	0.478^a^
CRP									
Day 0	8.4 (0.8–54.3)	16 (0.8–234.8)	**0.020** ^a^	12.4 (0.8–234.8)	10.7 (0.8–66.9)	0.698^a^	13.1 (0.8–234.8)	6.7 (0.8–62.5)	0.185^a^
Day 1	56 (15.2–183.5)	58.9 (15.2–222.2)	0.973^a^	57.5 (15.2–222.2)	58 (17–102.6)	0.599^a^	57.5 (15.2–222.2)	48 (17–102.3)	0.429^a^
Day 3	188.8 (102.9–322.7)	245.2 (78.2–383.5)	**0.003** ^a^	207.3 (78.2–383.5)	249 (113.6–354.1)	0.119^a^	207.3 (78.2–383.5)	249 (183–354.1)	0.060^a^
Day 7	56.5 (17.9–171.6)	127.4 (10.5–343.3)	**< 0.001** ^a^	79.8 (10.5–270)	150 (20.9–343.3)	**0.009** ^a^	84.1 (10.5–270)	135.4 (20.9–343.3)	0.109^a^
CRP change									
Day (0–1)	40.5 (2.5–157.6)	30 (− 84.5–175.4)	**0.013** ^a^	35.4 (− 84.5–175.4)	35.1 (− 41.6–84.3)	0.895^a^	35.4 (− 84.5–175.4)	36 (− 41.6–84.3)	0.794^a^
Day (0–3)	181.8 (82.2–301.2)	202.9 (− 8.1–368)	0.093^a^	185.7 (− 8.1–368)	241.3 (96.3–346.9)	0.093^a^	185.7 (− 8.1–368)	241.3 (146.1–346.9)	**0.036** ^a^
Day (0–7)	47.7 (− 14–169.2)	88 (− 123.6–336.1)	**0.013** ^a^	53.8 (− 123.6–224.9)	148 (10.6–336.1)	**0.004** ^a^	58 (− 123.6–224.9)	125.5 (10.6–336.1)	**0.049** ^a^
Day (1–3)	127.5 (32.8–262.4)	188.2 (9–351.1)	**0.008** ^a^	146.6 (9–351.1)	201.1 (82.3–335.2)	0.103^a^	146.6 (9–351.1)	201.1 (137.5–335.2)	**0.033** ^a^
Day (1–7)	−4.3 (− 137.9–139.2)	71.1 (− 177.2–324.4)	**< 0.001** ^a^	25.1 (− 177.2–192.6)	115.5 (− 46.1–324.4)	**0.008** ^a^	30.7 (− 177.2–192.6)	93.1 (− 46.1–324.4)	0.120^a^
Day (3–7)	−127.1 (− 201.5–−68.5)	−116.3 (− 256.1–52.9)	0.205^a^	−124.1 (− 256.1–16.4)	−86.1 (− 183.6–52.9)	0.095^a^	−123.8 (− 256.1–52.9)	−127.5 (− 183.6–−10.8)	0.821^a^
WBC									
Day 0	7.2 (3.8–15.7)	7.7 (3.4–16)	0.345^a^	7.4 (3.4–16)	8.1 (4.1–12.4)	0.471^a^	7.5 (3.4–16)	7.4 (5.1–12.4)	0.842^a^
Day 1	12.6 (4.8–22.8)	12.4 (3.2–36.1)	0.507^a^	12.4 (3.2–36.1)	14 (6.3–22.3)	0.449^a^	12.4 (3.2–36.1)	14 (6.3–22.3)	0.547^a^
Day 3	12.1 (6.9–19.9)	11.3 (4.7–30.5)	0.842^a^	11.8 (5.3–30.5)	12.1 (4.7–17.4)	0.794^a^	11.8 (5.3–30.5)	13.7 (4.7–16.5)	0.805^a^
Day 7	10.2 (6.1–15.7)	11 (5.4–22.4)	0.214^a^	10.4 (5.4–22.4)	11.8 (7.3–17.7)	**0.043** ^a^	10.4 (5.4–22.4)	11.8 (9–17.7)	0.070^a^
WBC change									
Day (0–1)	4.9 (− 1.6–18.8)	3.7 (− 1.6–27.1)	0.130^a^	4.1 (− 1.6–27.1)	5.1 (− 1.1–14.2)	0.427^a^	4.1 (− 1.6–27.1)	6.6 (− 1.1–14.2)	0.496^a^
Day (0–3)	4.5 (− 2.5–10.5)	3.9 (− 5.7–21.5)	0.559^a^	4 (− 5.7–21.5)	5 (− 3.4–9.7)	0.908^a^	4 (− 5.7–21.5)	5.9 (− 3.4–9.7)	0.679^a^
Day (0–7)	3.1 (− 3.2–7.6)	2.7 (− 6.3–18.2)	0.934^a^	2.6 (− 6.3–18.2)	5.4 (− 0.7–9.6)	**0.039** ^a^	2.7 (− 6.3–18.2)	5.4 (− 0.7–9.6)	0.064^a^
Day (1–3)	−1.4 (− 9.6–4.8)	0.1 (− 12–8.1)	0.184^a^	−0.1 (− 12–8.1)	−1.1 (− 8.3–4)	0.318^a^	−0.3 (− 12–8.1)	−1.3 (− 8.3–4)	0.453^a^
Day (1–7)	−2.8 (− 14–6.6)	−1.2 (− 23.8–10.2)	**0.043** ^a^	−1.5 (− 23.8–10.2)	−1.5 (− 11–5.1)	0.418^a^	−1.5 (− 23.8–10.2)	−2 (− 4.6–4.5)	0.881^a^
Day (3–7)	−2.1 (− 8.8–2.5)	−1.2 (− 18.2–12.8)	0.492^a^	−2.1 (− 18.2–12.8)	0.9 (− 3.9–6.2)	**0.008** ^a^	−1.8 (− 18.2–12.8)	0.9 (− 3.9–4.3)	**0.033** ^a^

*Abbreviations*: *CRP* C-reactive protein (mg/L), *MDW* Monocyte distribution width, *POPF* Postoperative pancreatic fistula, *WBC* White blood cells (× 10^9^/L)

Timepoint definitions: Day 0: preoperative day (before surgery); Day 1: postoperative day 1; Day 3: postoperative day 3; Day 7: postoperative day 7

Notes:

^a^Statistical test: Mann‒Whitney U test

Data are presented as the median (range)

Statistically significant p values (< 0.05) are highlighted in bold for clarity

All comparisons were made using the Mann‒Whitney U test

For pancreatic fistula, comparisons are between “No leak and biochemical leak” vs. “POPF B and C

The change values represent differences between the specified days (e.g., Day 0 − 1 = Day 1 value minus Day 0 value)

After Benjamini–Hochberg correction, MDW retained statistical robustness across several key outcomes. For overall complications, MDW remained significant on days 0, 1, 3, and 7 (q ≤ 0.03). For anastomotic leaks, MDW preserved significance at days 3 and 7 as well as over interval changes (0–3, 0–7, 1–3; q ≤ 0.047). For CR-POPF (grades B and C), MDW remained significant at days 3 and 7 and for changes over 1–3 and 1–7 (q ≤ 0.045).

In contrast, CRP retained significance in a narrower context—mainly at day 7 and a few change intervals—while most WBC-related comparisons lost significance after correction. These findings highlight MDW as the most consistent and reliable biomarker following appropriate adjustment for multiple testing. (Supplementary Tables S1a-c).

Machine learning analyses revealed that predictive performance was strongest for overall complications, with ROC-AUC values ranging from 0.72 to 0.83 and accuracies up to 0.88 (see Supplementary Table S2a). For anastomotic leaks, performance was modest (ROC-AUC 0.50–0.65; Supplementary Table S2b), while CR-POPF demonstrated limited discrimination (ROC-AUC 0.34–0.62; Supplementary Table S2c). SSI yielded intermediate results (ROC-AUC 0.51–0.76; Supplementary Table S2d). Across all analyses, MDW consistently contributed to the best-performing models, whereas CRP- and WBC-based models showed weaker and less stable predictive power.

### Surgical site infections

Dedicated analyses of SSI revealed significant associations between SSI incidence and several perioperative as well as laboratory variables. In univariate analyses, higher CD grade, preoperative biliary drainage, fresh frozen plasma (FFP) transfusion, lower preoperative albumin, and prolonged hospital stay were significantly related to SSI occurrence (Supplementary Table S3a). ROC curve evaluations demonstrated notable predictive performance for MDW on postoperative days 3 and 7, CRP on days 0, 3, and 7, and WBC on day 7 (Supplementary Table S3b). In multivariate logistic regression, low preoperative albumin, perioperative FFP transfusion, elevated MDW on day 7, and elevated CRP on day 7 emerged as independent predictors of SSI (Supplementary Table S3c).

### ROC and diagnostic test analysis results

ROC analysis demonstrated the superiority of the MDW over the CRP level and WBC count in the prediction of all postoperative complications. For general complications, the MDW showed progressively increasing diagnostic accuracy from day 0 (AUC 0.679, cut-off > 19.975) to day 7 (AUC 0.818, cut-off > 21.81), with peak performance observed on day 3 (AUC 0.777, cut-off > 23.1, all *p* < 0.001).

**Fig. 1:**
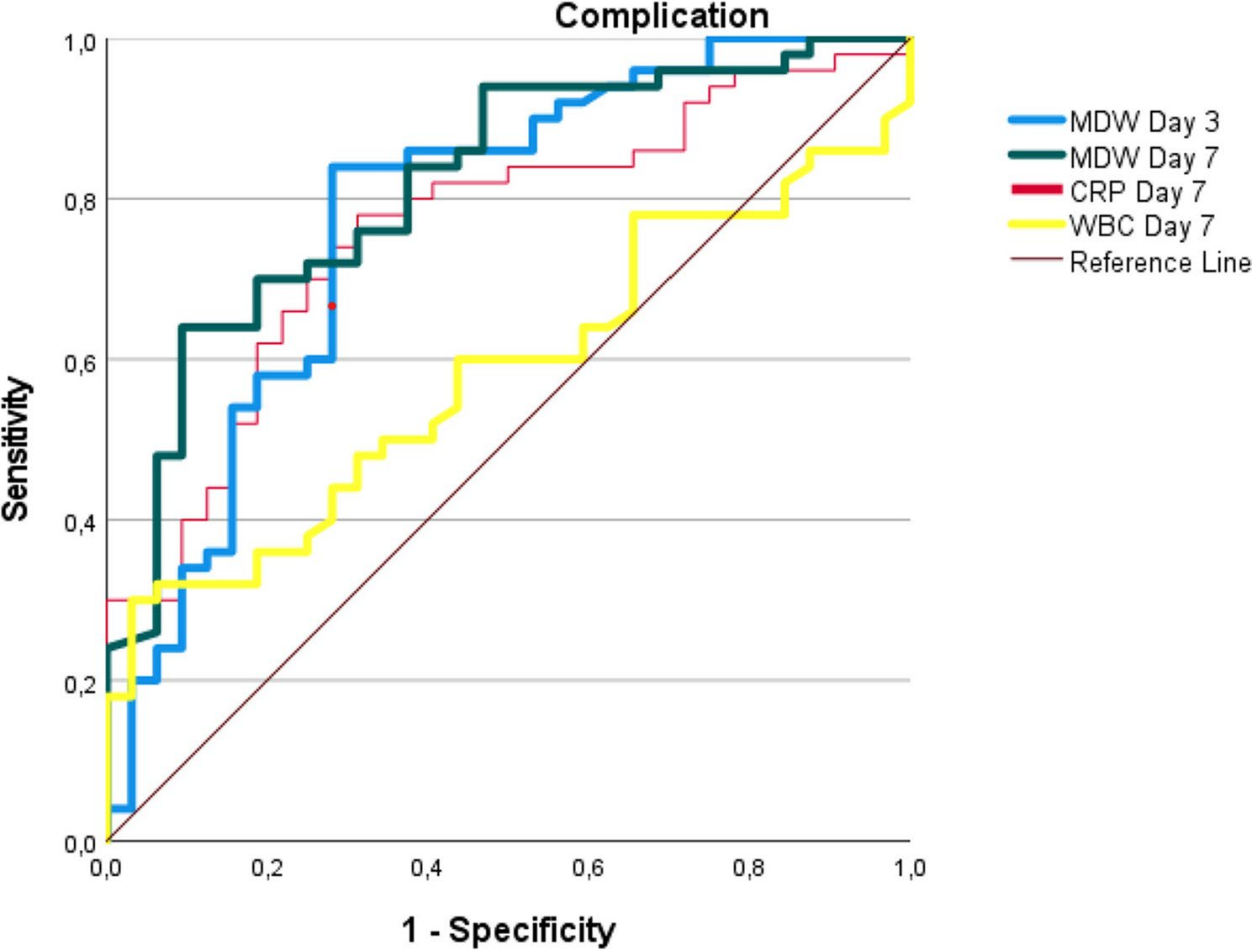
ROC curve analysis of MDW, CRP, and WBC for predicting postoperative complications. Figure Abbreviations (for ROC Curves):ROC: Receiver Operating Characteristic, MDW: Monocyte Distribution Width, CRP: C-Reactive Protein (mg/L), WBC: White Blood Cells (×10⁹/L)

**Fig. 2:**
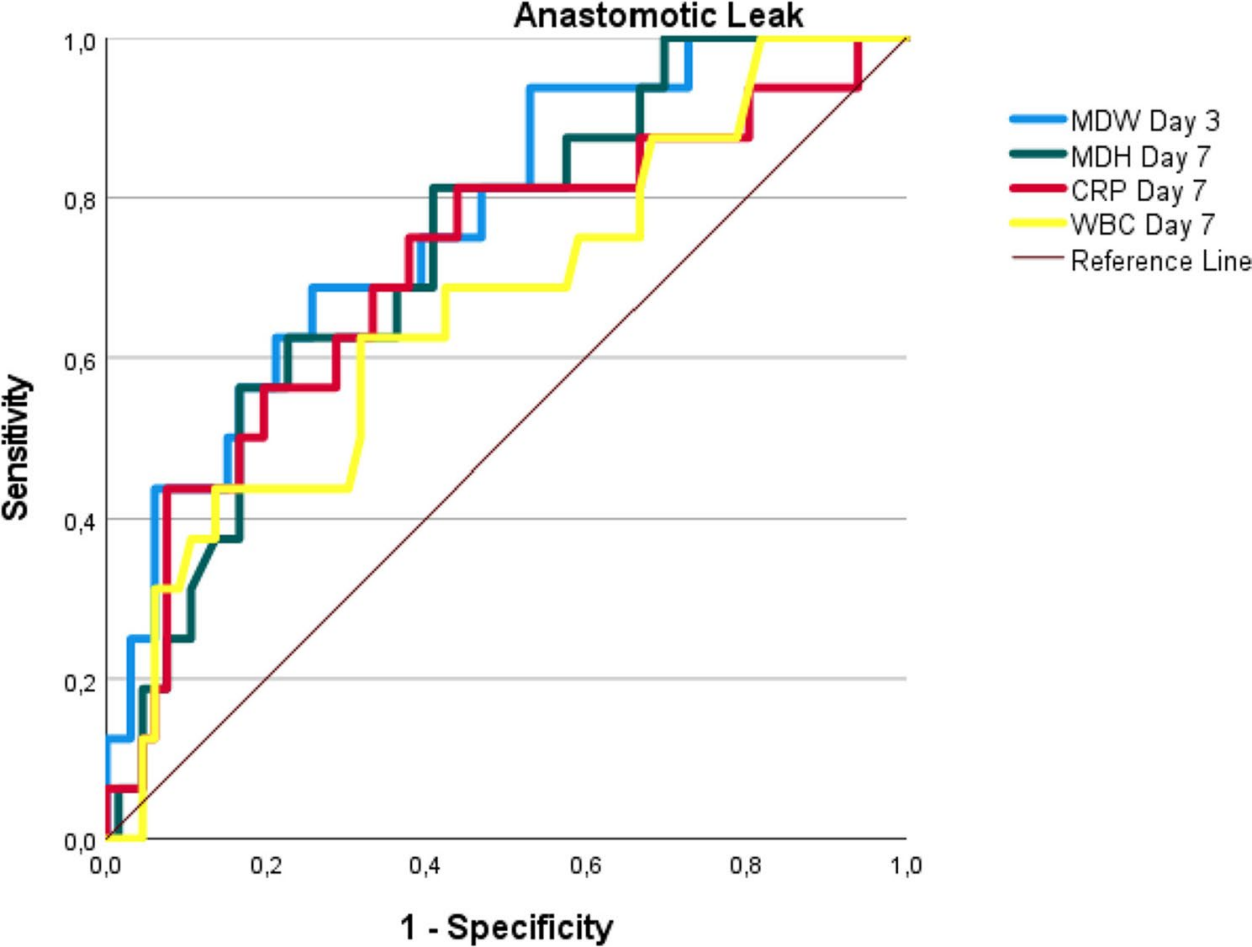
ROC curve analysis for anastomotic leak prediction. Figure Abbreviations (for ROC Curves): ROC: Receiver Operating Characteristic, MDW: Monocyte Distribution Width, CRP: C-Reactive Protein (mg/L), WBC: White Blood Cells (×10⁹/L)

**Fig. 3:**
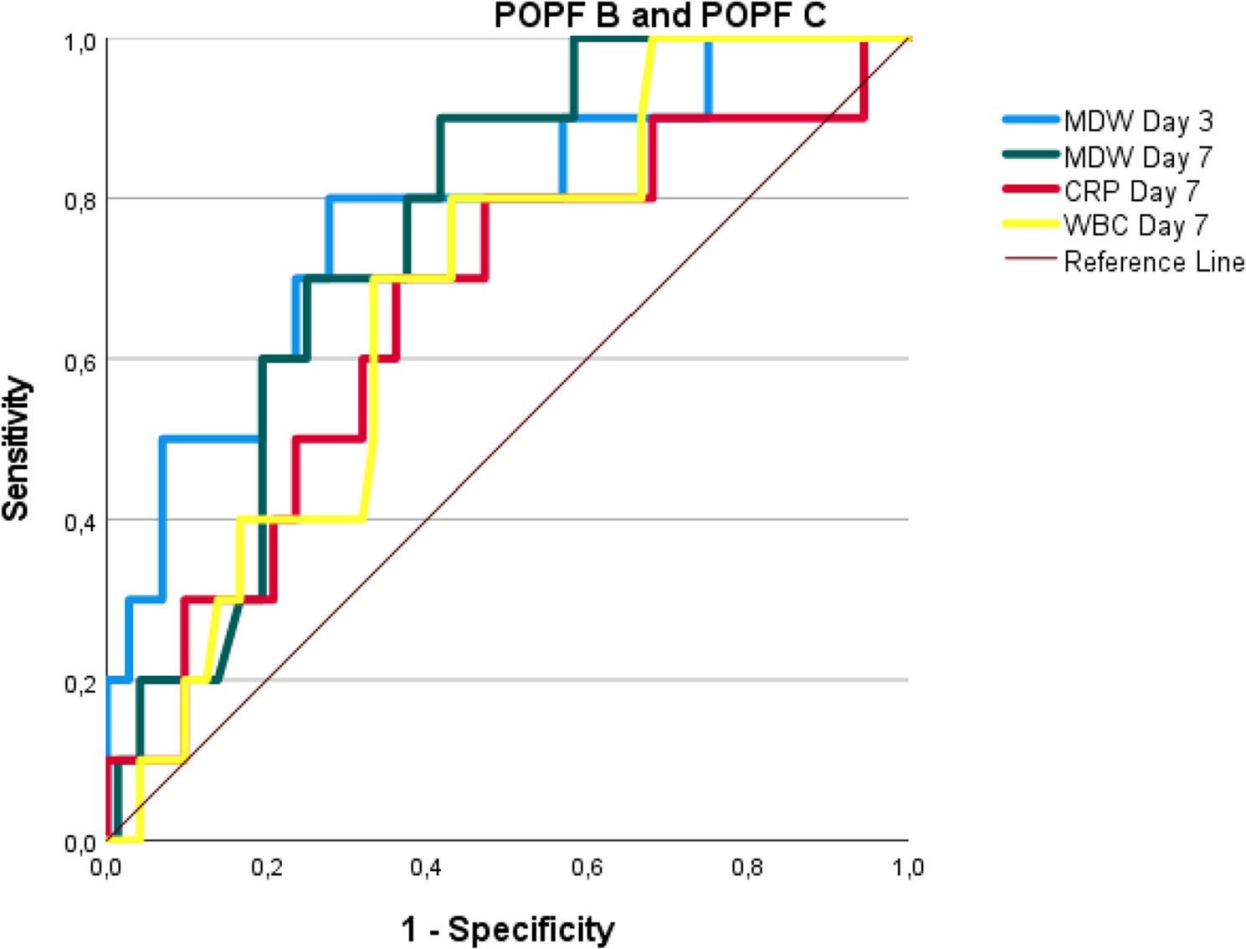
ROC curves for postoperative pancreatic fistula (POPF) prediction. Figure Abbreviations (for ROC Curves): ROC: Receiver Operating Characteristic, MDW: Monocyte Distribution Width, CRP: C-Reactive Protein (mg/L), WBC: White Blood Cells (×10⁹/L), POPF: Postoperative Pancreatic Fistula (Grade B/C)

In anastomotic leak detection, the MDW on day 3 (AUC 0.770, cut-off > 26.08) and day 7 (AUC 0.734, cut-off > 23) significantly outperformed the CRP level (AUC 0.711) and WBC count (AUC 0.662). Similarly, for pancreatic fistulas (POPF B/C), the MDW maintained excellent diagnostic capability on day 3 (AUC 0.781, cut-off> 26.0) and day 7 (AUC 0.758, cut-off > 23.5). Across all the measurements, the MDW demonstrated balanced sensitivity (77–80%) and specificity (62–79%) with clinically valuable likelihood ratios (+ LR 2.0–3.7), establishing it as the most reliable early warning biomarker when measured between postoperative days 3 and 7 (Table 4 and Figs. 1, 2 and 3).

**Table 4:** MDW, CRP, and WBC as predictors of postoperative complications, anastomotic leaks, and pancreatic fistulas

Variables	Cut off	Sensitivity	Specificity	+PV	−PV	+LR	−LR	AUC ± SE	*P* value
Complication									
MDW									
Day 0	> 19.975	68	65.6	75.6	56.8	2	0.4	0.679 ± 0.059	**0.006**
Day 1	> 21.070	64	65	64	53	2	0.5	0.677 ± 0.060	**0.003**
Day 3	> 23.145	84	72	82	74	2.9	0.2	**0.777 ± 0.055**	**< 0.001**
Day 7	> 21.81	84	62.5	78	71	2.24	0.2	**0.818 ± 0.047**	**< 0.001**
CRP									
Day 0	> 14.35	52	71	74	49	1.8	0.6	0.653 ± 0.060	**0.02**
Day 3	> 199.6	76	62.5	76	62	2	0.3	0.699 ± 0.058	**0.001**
Day 7	> 70	78	68	79	66.7	2.4	0.3	0.761 ± 0.053	**< 0.001**
CRP change									
Day (0–7)	> 54.7	68	65.6	75	56	2	0.4	0.666 ± 0.060	**0.011**
Day (1–3)	> 146.5	70	68.8	77	59	2.2	0.4	0.681 ± 0.060	**0.001**
Day (1–7)	> 36	68	75	81	60	2.72	0.4	0.741 ± 0.055	**< 0.001**
Anastomotic leak									
MDW									
Day 3	> 26.08	68.8	74.2	40	90.7	2.66	0.4	**0.770 ± 0.063**	**< 0.001**
Day 7	> 23	81.3	59.09	31	92	2	0.3	**0.734 ± 0.065**	**< 0.001**
CRP									
Day 7	> 98.45	75	60	31	90	2	0.4	0.711 ± 0.077	**0.006**
WBC									
Day 7	> 11.6	62.5	68.2	32	88.2	2	0.55	0.662 ± 0.077	**0.035**
MDW change									
Day (0–3)	> 3.6	93	53	31	97	2	0.12	0.738 ± 0.072	**< 0.001**
Day (0–7)	> 4.4	60	75.8	36	90	2.4	0.5	0.703 ± 0.072	**0.004**
CRP change									
Day (0–7)	> 86	68	65.2	32	90	2	0.4	0.731 ± 0.073	**0.001**
Day (1–7)	> 71	62.5	68.2	32	88	2	0.55	0.709 ± 0.076	**0.006**
WBC change									
Day (3–7)	> 0	62	72	35	88	2	0.6	0.718 ± 0.069	**0.007**
Day (0–7)	> 3.7	68	68	34	90	2.1	0.45	0.667 ± 0.088	**0.039**
POPF B and POPF C									
MDW									
Day 3	> 26.0	80	72	27	96	2.8	0.3	**0.781 ± 0.081**	**0.004**
Day 7	> 23.5	77	62	23	95	2	0.3	**0.758 ± 0.064**	**0.008**
Day (1–3)	> 5.0	77	79	32	96.6	3.7	0.28	0.758 ± 0.103	**0.012**
Day (1–7)	> 2.5	78	66	22.6	96	2.3	0.3	0.728 ± 0.098	**0.027**
CRP									
Day (0–3)	> 232	70	70	24	94	2.5	0.3	0.737 ± 0.77	**0.021**
Day (0–7)	> 106	60	70	21	92	2.1	0.48	0.750 ± 0.074	**0.015**
Day (1–3)	> 193	60	66.7	20	92	2	0.5	0.721 ± 0.070	**0.032**

*Abbreviations*: +*LR* Positive likelihood ratio,,−*LR* Negative likelihood ratio, *AUC* Area under the ROC curve, *CRP* C-reactive protein (mg/L), *MDW* Monocyte distribution width, *p* *p* value (statistical significance), *POPF* Postoperative pancreatic fistula (Grade B/C), +*PV* Positive predictive value, -*PV* Negative predictive value, *Preop* Preoperative (day 0), *SE* Standard error; WBC: white blood cells (× 10^9^/L)

Timepoint definitions: Day 0: preoperative day (before surgery); Day 1: postoperative day 1; Day 3: postoperative day 3; Day 7: postoperative day 7

The change values represent differences between the specified days (e.g., Day 0 − 1 = Day 1 value minus Day 0 value)

Notes:

Statistically significant *p* values (< 0.05) are highlighted in bold for clarity

These results suggest that the MDW has the potential to become a standard monitoring parameter for predicting surgical complications before clinical manifestations.

### Correlation analysis results

Early Predictive Value of MDW for Subsequent Inflammatory Responses Correlation analysis revealed that MDW levels on postoperative day 3, which demonstrated superior diagnostic performance in ROC analyses, were significantly positively correlated with key inflammatory markers measured later in recovery. The strongest correlation emerged between the day 3 and day 7 MDW values (r = 0.587, *p* < 0.001), indicating consistent monocyte activation patterns throughout the critical postoperative week. Importantly, the MDW on day 3 also correlated significantly with the CRP level on day 7 (r = 0.468, *p* < 0.001) and WBC count on day 7 (r = 0.270, *p* = 0.014), demonstrating its ability to predict the subsequent systemic inflammatory response typically captured by these conventional markers (Table 5).

**Table 5:** Correlation of pre- and postoperative MDW, CRP, and WBC levels on days 0, 1, 3, 7

Variables	MDW Day								CRP Day								WBC Day					
	0		1		3		7		0		1		3		7		0		1		7	
	r	*P* value	r	*P* value	r	*P* value	r	*P* value	r	*P* value	r	*P* value	r	*P* value	r	*P* value	r	*P* value	r	*P* value	r	*P* value
MDW Day																						
1	0.282	**0.010**	–																			
3	0.28	**0.011**	0.3	**0.006**	–																	
7	0.307	**0.005**	0.399	**< 0.001**	0.587	**< 0.001**	–															
CRP Day																						
0	0.423	**< 0.001**	0.170	0.126	0.153	0.171	0.207	0.061	–													
1	0.269	**0.015**	0.179	0.107	0.007	0.951	0.114	0.308	0.492	**< 0.001**	–											
3	0.213	0.054	0.196	0.077	0.342	**0.002**	0.346	**0.001**	0.124	0.269	0.201	0.070	–									
7	0.142	0.202	0.222	**0.045**	0.468	**< 0.001**	0.607	**< 0.001**	0.135	0.225	0.095	0.398	0.692	**0.000**	–							
WBC Day																						
0	0.123	0.270	0.036	0.745	0.312	**0.004**	0.273	**0.013**	0.117	0.293	0.213	0.055	0.180	0.105	0.179	0.108	–					
1	0.069	0.539	0.198	0.075	0.002	0.985	0.058	0.602	0.148	0.184	0.055	0.623	0.110	0.326	0.087	0.436	0.435	**< 0.001**	–			
3	0.040	0.720	0.144	0.198	0.083	0.460	0.003	0.982	0.119	0.287	−0.094	0.403	0.088	0.432	0.063	0.574	0.485	**< 0.001**	0.624	**< 0.001**	–	
7	0.111	0.321	0.043	0.700	0.270	**0.014**	0.26	**0.018**	0.186	0.094	0.130	0.246	0.254	**0.021**	0.257	**0.020**	0.462	**< 0.001**	0.438	**< 0.001**	0.567	**< 0.001**

*Abbreviations*: *CRP* C-reactive protein (mg/L), *MDW* Monocyte distribution width, *r*: Correlation coefficient (Pearson/Spearman), *WBC* White blood cells (×10⁹/L)

Timepoint definitions: Day 0: preoperative day (before surgery); Day 1: postoperative day 1; Day 3: postoperative day 3; Day 7: postoperative day 7

Notes:

Statistically significant *p* values (< 0.05) are highlighted in bold for clarity

These findings collectively suggest that elevated MDW values as early as postoperative day 3 may reliably predict the developing inflammatory state that becomes fully apparent by day 7, potentially enabling earlier clinical intervention.

### Multivariate logistic regression analysis

Multivariate logistic regression analysis revealed that the MDW is an independent predictor of multiple postoperative complications. For overall complications, MDW on postoperative day 7 was identified as an independent predictor (OR 1.479, 95% CI 1.113–1.965; *p* = 0.007), together with CRP on day 7 (OR 1.015; *p* = 0.015), transfusion requirements (OR 1.279; *p* = 0.043), male gender (OR 7.217; *p* = 0.018), and low preoperative albumin (OR 11.499; *p* = 0.002). In relation to anastomotic leaks, the change in MDW between days 0–3 was significantly associated with leak development (OR 1.299; *p* = 0.015), along with pancreatic duct diameter (OR 2.217; *p* = 0.016) and red blood cells (RBC) transfusion (OR 1.515; *p* = 0.021). For CR-POPF (grades B and C), MDW on day 3 was an independent predictor (OR 1.31; *p* = 0.044), in addition to pancreatic duct diameter (OR 35.305; *p* = 0.005) and RBC transfusion (OR 2.482; *p* = 0.008). Furthermore, Cox regression analysis showed that MDW values on postoperative days 0 and 1 were significantly associated with overall survival (HR 1.278; *p* = 0.038 and HR 1.459; *p* < 0.001), together with CRP on day 0 (HR 1.034; *p* < 0.001), FFP transfusion (HR 1.597; *p* < 0.001), and comorbidity (HR 4.041; *p* = 0.043). Taken together, these results highlight that MDW was the only inflammatory biomarker consistently retained as an independent predictor across multiple outcomes, including overall complications, anastomotic leaks, CR-POPF, and survival. In contrast, conventional markers such as CRP and WBC either lost significance or showed weaker associations in multivariate models. This consistency underscores the potential role of MDW as an earlier and more reliable predictor compared with traditional parameters.

Post-hoc power analyses were conducted to further evaluate the robustness of these regression models. For overall complications, the effect of MDW on postoperative day 7 (b = 0.39, SE = 0.14; OR 1.48; *p* = 0.007) achieved a power of ≈ 0.80, indicating adequate sensitivity to detect this association within the current dataset. In contrast, the predictive effects for anastomotic leaks (b = 0.26, SE = 0.11; OR 1.30; *p* = 0.015; power ≈ 0.66) and CR-POPF (b = 0.27, SE = 0.13; OR 1.31; *p* = 0.044; power ≈ 0.55) demonstrated only moderate or limited sensitivity, largely due to the low number of observed events. These findings suggest that, although biologically and clinically plausible, the results should be regarded as hypothesis-generating rather than confirmatory, underscoring the need for validation in larger, multi-center cohorts.

## Discussion

To our knowledge, no prior study has investigated the relationship between the MDW and postoperative complications following pancreatic surgery. Only one study compared MDW with CRP level and WBC count and concluded that MDW more accurately identified complicated diverticulitis [12]. The majority of MDW-focused research has focused on its role in the detection of systemic infections, particularly sepsis and COVID-19 [9–11, 13, 14].

In this study, we compared the diagnostic performance of the MDW with that of the CRP level and WBC count for the prediction of postoperative complications following PD. The discussion will proceed by first evaluating MDW's predictive accuracy for overall complications, followed by anastomotic leaks, and subsequently, its role in identifying CR-POPF.

The ROC analysis demonstrated that the MDW on postoperative day 7 had the highest diagnostic performance for overall complications, with an AUC of 0.818 ± 0.047 (*p* < 0.001), surpassing that of both the CRP level and the WBC count. Furthermore, the day 3 MDW value also showed robust predictive ability (AUC: 0.777 ± 0.055, *p* < 0.001) (Table 4 and Fig. 1). At a cut-off of 23.14 on day 3, the MDW demonstrated a sensitivity of 84%, specificity of 72%, and notable NPV of 74%, making it the most reliable diagnostic tool among the evaluated parameters.

Multivariate logistic regression confirmed the independent predictive power of the MDW. A one-unit increase in MDW on day 7 was associated with a 1.49-fold increase in the risk of complications (*p* = 0.007, OR: 1.479; CI: 1.113–1.965), which was substantially greater than that the value associated with the CRP level (*p* = 0.015, OR: 1.015; CI: 1.003–1.028) (Table 6).

**Table 6:** Multivariate logistic regression and Cox-regression analyses of postoperative complications, anastomotic leaks, pancreatic fistula

Variables	B (SE)	*P* value	Odds ratio (95% CI)
Multiple logistic regression analysis results			
Complication			
Age	0.03 (0.03)	0.303	1.031 (0.973–1.093)
Sex (Male)	1.98 (0.84)	**0.018**	7.217 (1.403–37.13)
Preop albumin (↓)	2.44 (0.8)	**0.002**	11.499 (2.38–55.563)
Number of FFP transfusions^*^ (↑)	0.25 (0.12)	**0.043**	1.279 (1.007–1.624)
MDW day 7 (↑)	0.39 (0.14)	**0.007**	1.479 (1.113–1.965)
CRP day 7 (↑)	0.02 (0.01)	**0.015**	1.015 (1.003–1.028)
Percentage correct			
Specificity	71.9		
Sensitivity	86		
Overall	80.5		
Anastomotic leak			
Age	0 (0.02)	0.903	1.003 (0.962–1.045)
Sex (Male)	1.11 (0.79)	0.160	3.041 (0.644–14.357)
Pancreatic duct diameter, mm (↓)	0.8 (0.33)	**0.016**	2.217 (1.161–4.233)
Number of RBC transfusion^*^ (↑)	0.42 (0.18)	**0.021**	1.515 (1.064–2.158)
MDW change (0–3) (↑)	0.26 (0.11)	**0.015**	1.299 (1.052–1.605)
Percentage correct			
Specificity	98.5		
Sensitivity	56.3		
Overall	90.2		
POPF B and POPF C			
Age	0.01 (0.05)	0.883	1.007 (0.92–1.102)
Sex (Male)	0.16 (1.01)	0.878	1.168 (0.161–8.499)
Pancreatic duct diameter, mm (↓)	3.56 (1.27)	**0.005**	35.305 (2.913–427.823)
Number of RBC transfusions^*^ (↑)	0.91 (0.35)	**0.008**	2.482 (1.262–4.881)
MDW day 3 (↑)	0.27 (0.13)	**0.044**	1.31 (1.007–1.705)
Percentage correct			
Specificity	97.2		
Sensitivity	70.0		
Overall	93.9		
COX regression analysis results on survival			
MDW day 0 (↑)	**0.118**	**0.038**	1.278 (1.013–1.1610)^a^
MDW day 1 (↑)	**0.090**	**< 0.001**	1.459 (1.224–1.739)^a^
CRP day 0 (↑)	**0.090**	**< 0.001**	1.034 (1.016–1.053)^a^
Number of FFP transfusions^*^ (↑)	**0.092**	**< 0.001**	1.597 (1.335–1.911)^a^
Comorbidity	**0.691**	**0.043**	4.041 (1.043–15.655)^a^

*Abbreviation*: *b* Regression coefficient, *CI* Confidence interval, *CRP* C-reactive protein (mg/L), *FFP* Fresh frozen plasma, *MDW* Monocyte distribution width, *POPF* Postoperative pancreatic fistula, *Pre-op* Preoperative, *RBC* Red blood cells, *SE* Standard error, *WBC* White blood cells (×10⁹/L)

Notes:

^a^ Indicates Hazard ratio (95% CI)

Statistically significant *p* values (< 0.05) are highlighted in bold for clarity

The arrows indicate the direction of association: (↑) = Increased value associated with higher risk. (↓) = Decreased value associated with higher risk

*Blood product counts reflect transfusions within 7 postoperative days

When focusing specifically on anastomotic leaks, the MDW on day 3 again had the highest diagnostic value (AUC: 0.770 ± 0.063, *p* < 0.001), outperforming the CRP level and WBC count. With a cut-off value of 26.08, the sensitivity was 68%, the specificity was 74.2%, and the NPV reached 90% (Table 4 and Fig. 2). Furthermore, a one-unit increase in the MDW from day 0 to day 3 was associated with a 1.2-fold increased risk of anastomotic leak (*p* = 0.015, OR: 1.299; CI: 1.052–1.605), indicating that the MDW is not only a diagnostic tool but also a prognostic indicator (Table 6).

In the literature, POPF remains the most frequently studied complication following pancreatic surgery [2, 15–18]. The CRP level, WBC count, postoperative drain amylase level, and procalcitonin (PCT) level have all been evaluated as predictive markers for POPF [19–26]. The CRP level has been widely studied and reported to be significant in this context [19–21]. PCT has also been highlighted for its diagnostic value [20, 24, 25]. Caputo et al. [27] investigated other haematological markers, such as the neutrophil‒lymphocyte ratio and WBC count, alongside drain amylase values, for POPF diagnosis.

Despite these efforts, the current diagnostic markers have limitations. Drain amylase, for example, is affected by centre-dependent variability in drain placement, the amount of remaining pancreatic tissue, and the lack of correlation with clinical progression [21, 27, 28]. Diagnostic studies are ongoing to identify better predictors, including analyses of bile duct flora and presepsin levels in blood and drain fluid [22, 26].

In our cohort, the MDW had a statistically significant predictive value for CR-POPF. The highest AUC was recorded for MDW on day 3 (AUC: 0.781 ± 0.081, *p* = 0.004), followed by day 7 (AUC: 0.758 ± 0.064, *p* = 0.008) (Table 4 and Fig. 3). At a cut-off of 26 on day 3, the MDW exhibited a sensitivity of 80%, a specificity of 72%, and an exceptionally high NPV of 96%. Multivariate analysis revealed that MDW was the only inflammatory marker independently associated with CR-POPF (*p* = 0.044, OR: 1.31; CI: 1.007–1.705) (Table 6). Additionally, a change of more than 5 units in the MDW between preoperative day 0 and day 3 was highly significant for predicting POPF (Table 4).

Our analyses demonstrated that MDW consistently outperformed CRP and WBC. Importantly, even after applying the Benjamini–Hochberg FDR method—appropriate for serially collected, correlated biomarkers—MDW retained its predictive strength. In particular, MDW on postoperative day 3 remained independently and significantly associated with overall complications (*p* < 0.001, q = 0.004), anastomotic leaks (*p* = 0.001, q = 0.006), and CR-POPF (*p* = 0.003, q = 0.010), whereas most CRP- and WBC-related signals did not withstand correction (Supplementary Tables S1). These results emphasize that among perioperative markers, MDW provides the earliest and most reliable warning signal. The robustness of MDW after rigorous correction further supports its clinical utility as a routine biomarker for guiding early intervention in the postoperative period.

Machine learning findings further support our central observation that MDW is the most reliable early biomarker after pancreatic surgery. The strong performance for overall complications, with cross-validated ROC-AUCs reaching 0.83, highlights the reproducibility of MDW’s predictive signal even under conservative evaluation. By contrast, the weaker discrimination for anastomotic leaks and CR-POPF likely reflects the limited number of events and class imbalance, rather than a lack of true association (Supplementary Tables S2). Taken together, the concordance between univariate analyses and machine learning results underscores MDW’s superiority over CRP and WBC, reinforcing its potential as a clinically actionable biomarker.

Importantly, MDW remains a relatively novel biomarker, and no universal or standardized cut-off values have yet been established in the surgical literature. Therefore, our study represents one of the first attempts to define clinically relevant thresholds for MDW in the context of pancreatic surgery. We consider this exploratory nature a strength, as it provides a foundation for future research to confirm, refine, and potentially standardize MDW cut-off values across diverse clinical settings. While the identified thresholds provide useful preliminary insights, their external validation in larger, multicenter prospective cohorts will be essential before routine clinical application.

In previous studies, CRP has been widely utilized as a reliable inflammatory marker and has demonstrated significant associations with postoperative complications, including CR-POPF [19–21, 29, 30]. However, in our study, MDW outperformed CRP not only in diagnostic accuracy but also in its ability to provide earlier indications of inflammatory processes. This earlier response highlights MDW’s potential as a more sensitive and timely marker in the immediate postoperative period, offering a meaningful advantage in clinical decision-making and complication monitoring.

Notably, although the positive predictive values of the MDW were modest, its high NPV suggest that it could be extremely useful in clinical decision-making. Specifically, it may help identify patients at low risk for complications, enabling earlier drain removal or oral intake initiation. This can also help reduce unnecessary imaging and interventions.

On the other hand, in patients clinically suspected of having complications and with MDW above the cut-off, further diagnostic imaging or therapeutic interventions may be justified.

Some studies have proposed the use of somatostatin or its analogues as a therapeutic strategy to reduce the incidence or severity of CR-POPF [31–33]. However, the timing of intervention and the identification of patients who would truly benefit from such treatment remain unresolved challenges. This uncertainty largely stems from the difficulty in accurately predicting when a leak will occur and in which patients. Our study suggests that MDW, by enabling earlier identification of inflammatory changes associated with anastomotic leaks, may help to narrow this therapeutic window. With the more precise estimation of the onset of CR-POPF provided by MDW trends—particularly the significant elevations observed around postoperative day 3—the administration of somatostatin analogues could be timed more effectively. This may enhance both the clinical utility and the scientific rationale for incorporating somatostatin-based therapies into future protocols or interventional studies targeting CR-POPF management.

Survival analyses in our study demonstrated that MDW values on postoperative days 0 and 1, along with CRP levels on day 0, were significantly associated with patient outcomes according to Cox regression analysis (Table 6). These findings suggest a potential prognostic role for MDW in the postoperative course. However, importantly, MDW remains a relatively novel biomarker, and our study was conducted with a limited follow-up duration, which may limit the ability to fully elucidate its long-term prognostic implications. While statistical associations with survival were observed, we refrained from emphasizing prognostic conclusions due to the inherent limitations of our dataset. In contrast to CRP and cachexin levels, which have been more extensively studied in relation to the systemic immune response and pancreatic cancer prognosis [34], the role of MDW in long-term oncological outcomes remains largely unexplored. Prospective studies with extended follow-up periods are needed to more definitively assess the prognostic value of MDW in this context.

Perhaps one of the most compelling findings of our study is the potential of the MDW as an early warning marker—a “canary in the coal mine”—in the context of postoperative complications. While CRP and WBC elevations may lag behind clinical symptoms, the MDW appears to rise earlier, potentially offering clinicians a valuable window for earlier intervention. Correlation analysis further supported the significant relationships among the MDW, CRP level, and WBC count (Table 5), reinforcing MDW’s central role in the postoperative inflammatory cascade.

In addition to our findings, it is important to contextualize MDW against conventional biomarkers. Numerous studies have examined CRP, PCT and WBC as predictors of postoperative complications after pancreatic surgery. CRP typically rises within 24–72 h, but its specificity is limited and false positives are common [19, 25, 29]. PCT demonstrates higher specificity, but its measurement is costly and not universally available [24, 25]. Drain amylase, although widely used, has center-dependent variability and limited correlation with clinical progression [20, 21, 27, 28]. Prospective analyses confirm that although CRP and PCT correlate with severe complications, neither consistently identifies early POPF when assessed on postoperative day 1 [19, 25]. By contrast, MDW is obtained from routine complete blood counts at no additional cost and has demonstrated earlier and more consistent diagnostic performance across multiple studies [6, 9–11, 19, 24, 25]. In our cohort, postoperative day 3 MDW emerged as an independent predictor of both overall complications and CR-POPF, whereas CRP and WBC did not retain predictive value in multivariate analysis, further supporting its clinical utility.

The pathophysiological basis of MDW lies in monocyte activation and morphological heterogeneity during systemic inflammation. Circulating histones, cytokines, and pathogen-associated molecular patterns rapidly trigger monocyte swelling, vacuolization, and nuclear remodeling, changes that are captured as increased MDW by automated hematology analyzers [6, 7]. Experimental data indicate that MDW can increase within hours of exposure to inflammatory stimuli, preceding measurable rises in CRP or WBC [5, 6]. This early sensitivity reflects the biological rationale for MDW as a superior biomarker for surgical complications.

Another strength of MDW is its practicality—it is readily available through routine complete blood count tests at no additional cost, and provides rapid results. This distinguishes it from markers such as PCT, which are costlier to measure and not always available in routine practice. Indeed, PCT was not evaluated in our study because of its limited use in our centre, and prior studies have noted its cost disadvantages [24, 25].

Despite these promising findings, our study has certain limitations. This was a retrospective, single-centre analysis, which may limit the generalizability of our results.

The small number of clinically relevant POPF events (*n* = 10) constrained statistical power; post-hoc analysis indicated a power of only ~ 0.55 for MDW on day 3. To achieve adequate power (~ 80%), approximately 150 patients with 18–20 CR-POPF events would be required. Survival analyses also suggested a possible prognostic role for MDW, but short follow-up and modest sample size preclude firm conclusions. Finally, although variables used in fistula risk models were available, we did not calculate the Fistula Risk Score (FRS) or the Alternative Fistula Risk Score (a-FRS) to preserve the primary focus on MDW. Future multicenter studies with larger cohorts and extended follow-up are needed to confirm these findings and to assess the additive value of MDW alongside established risk models.

## Conclusions

MDW appears to be a promising biomarker for predicting postoperative complications, including anastomotic leaks and POPF, after PD. By reflecting early inflammatory changes, it may enable timelier recognition of critical events compared with conventional markers. Its availability through routine blood counts also offers a practical and cost-free advantage. However, given the retrospective design and limited sample size of this study, these findings should be considered exploratory. Larger prospective cohorts are required to validate the predictive role of MDW and to define its potential integration into perioperative risk stratification and postoperative monitoring.

## Supplementary Information

Supplementary Material 1

Supplementary Material 2

Supplementary Material 3

Supplementary Material 4

## Data Availability

This study included preoperative and postoperative biochemical and numerical data from patients who underwent PD. Images and tissue samples from the patients were not used. Patient data are anonymized. These data are not publicly available. However, we can share anonymized patient data upon request (Email: sakaoglub@gmail.com).

## Abbreviations

AUC: Area under the (ROC) curve
a-FRS: Alternative fistula risk score
CD: Clavien–Dindo
CI: Confidence interval
CRP: C-reactive protein (mg/L)
CR-POPF: Clinically relevant postoperative pancreatic fistula
ERCP: Endoscopic retrograde cholangiopancreatography
FDA: Food and Drug Administration
FDR: False discovery rate
FFP: Fresh frozen plasma
FRS: Fistula risk score
HR: Hazard ratio
ISGLS: International Study Group for the Surgery of the Liver
ISGPS: International Study Group of Pancreatic Surgery
k-NN: k-nearest neighbors
KVKK: Kişisel Verilerin Korunması Kanunu (Turkey’s Personal Data Protection Law)
LDA: Linear discriminant analysis
MDW: Monocyte distribution width
NPV: Negative predictive value
OR: Odds ratio
PBD: Percutaneous biliary drainage
PCT: Procalcitonin
PD: Pancreaticoduodenectomy
PDS: Polydioxanone (suture)
PG: Polyglyconate (suture)
POPF: Postoperative pancreatic fistula
PPV: Positive predictive value
QDA: Quadratic discriminant analysis
RBC: Red blood cell(s)
RBF: Radial basis function
ROC: Receiver operating characteristic
ROC-AUC: Area under the ROC curve
SE: Standard error
SSI: Surgical site infection
SVM: Support vector machine
VCS: Volume, conductivity, scatter
WBC: White blood cell(s) (×10⁹/L)
