# Supplementary material for: Evaluation of monocyte distribution width as a predictive factor for early complications of pancreatic surgery (pancreaticoduodenectomy): a retrospective cohort study
Source: BMC Surg. 2025 Nov 3;25:518. doi: 10.1186/s12893-025-03272-2 (PMC12581569; doi:10.1186/s12893-025-03272-2)
Supplement: Supplementary file 1 — Supplementary Material 1 [file 12893_2025_3272_MOESM1_ESM.docx]

**Supplementary Table S1a. Benjamini–Hochberg FDR results for overall complications**

| Marker | Measure | P | Q_BH | Significant_q<0.05 |
| --- | --- | --- | --- | --- |
| CRP | Change 0–1 | **0.013** | **0.039** | True |
| CRP | Change 0–3 | 0.093 | 0.199 | False |
| CRP | Change 0–7 | **0.013** | **0.039** | True |
| CRP | Change 1–3 | **0.008** | **0.030** | True |
| CRP | Change 1–7 | **0.001** | **0.007** | True |
| CRP | Change 3–7 | 0.205 | 0.321 | False |
| CRP | Day 0 | **0.020** | 0.055 | False |
| CRP | Day 1 | 0.973 | 0.973 | False |
| CRP | Day 3 | **0.003** | **0.018** | True |
| CRP | Day 7 | **0.001** | **0.007** | True |
| MDW | Change 0–1 | 0.879 | 0.966 | False |
| MDW | Change 0–3 | 0.285 | 0.407 | False |
| MDW | Change 0–7 | 0.142 | 0.256 | False |
| MDW | Change 1–3 | 0.145 | 0.256 | False |
| MDW | Change 1–7 | 0.090 | 0.199 | False |
| MDW | Change 3–7 | 0.924 | 0.966 | False |
| MDW | Day 0 | **0.007** | **0.030** | True |
| MDW | Day 1 | **0.008** | **0.030** | True |
| MDW | Day 3 | **0.001** | **0.007** | True |
| MDW | Day 7 | **0.001** | **0.007** | True |
| WBC | Change 0–1 | 0.130 | 0.256 | False |
| WBC | Change 0–3 | 0.559 | 0.671 | False |
| WBC | Change 0–7 | 0.934 | 0.966 | False |
| WBC | Change 1–3 | 0.184 | 0.307 | False |
| WBC | Change 1–7 | **0.043** | 0.107 | False |
| WBC | Change 3–7 | 0.492 | 0.634 | False |
| WBC | Day 0 | 0.345 | 0.470 | False |
| WBC | Day 1 | 0.507 | 0.634 | False |
| WBC | Day 3 | 0.842 | 0.966 | False |
| WBC | Day 7 | 0.214 | 0.321 | False |

Abbreviations: CRP: C-reactive protein (mg/L); MDW: monocyte distribution width; Q_BH: Benjamini–Hochberg adjusted q-value; WBC: white blood cells (× 10^9^/L).

Timepoint definitions: Day 0: preoperative day (before surgery); Day 1: postoperative day 1; Day 3: postoperative day 3; Day 7: postoperative day 7.

The change values represent differences between the specified days (e.g., Day 0−1 = Day 1 value minus Day 0 value).

Values are presented with three decimals. Statistical significance after Benjamini–Hochberg correction is indicated in the last column (q<0.05).

Notes:Statistically significant p values (< 0.05) are highlighted in bold for clarity.

**Supplementary Table S1b. Benjamini–Hochberg FDR results for anastomotic leak outcomes**

| Marker | Measure | P | Q_BH | Significant_q<0.05 |
| --- | --- | --- | --- | --- |
| CRP | Change 0–7 | **0.001** | **0.004** | True |
| CRP | Change 1–7 | **0.006** | **0.011** | True |
| CRP | Day 7 | **0.009** | **0.012** | True |
| MDW | Change 0–3 | **0.001** | **0.004** | True |
| MDW | Change 0–7 | **0.004** | **0.009** | True |
| MDW | Change 1–3 | **0.047** | **0.047** | True |
| MDW | Day 3 | **0.001** | **0.004** | True |
| MDW | Day 7 | **0.004** | **0.009** | True |
| WBC | Change 0–7 | **0.039** | **0.047** | True |
| WBC | Change 3–7 | **0.008** | **0.012** | True |
| WBC | Day 7 | **0.043** | **0.047** | True |

Abbreviations: CRP: C-reactive protein (mg/L); MDW: monocyte distribution width; Q_BH: Benjamini–Hochberg adjusted q-value; WBC: white blood cells (× 10^9^/L).

Timepoint definitions: Day 0: preoperative day (before surgery); Day 1: postoperative day 1; Day 3: postoperative day 3; Day 7: postoperative day 7.

The change values represent differences between the specified days (e.g., Day 0−1 = Day 1 value minus Day 0 value).

Values are presented with three decimals. Statistical significance after Benjamini–Hochberg correction is indicated in the last column (q<0.05).

Notes:Statistically significant p values (< 0.05) are highlighted in bold for clarity.

**Supplementary Table S1c. Benjamini–Hochberg FDR results for POPF B/C outcomes**

| Marker | Measure | P | Q_BH | Significant_q<0.05 |
| --- | --- | --- | --- | --- |
| CRP | Change 0–3 | **0.036** | 0.068 | False |
| CRP | Change 0–7 | **0.049** | 0.082 | False |
| CRP | Change 1–3 | **0.033** | 0.068 | False |
| CRP | Change 1–7 | 0.120 | 0.139 | False |
| CRP | Change 3–7 | 0.821 | 0.821 | False |
| CRP | Day 3 | 0.060 | 0.090 | False |
| CRP | Day 7 | 0.109 | 0.136 | False |
| MDW | Change 0–3 | **0.024** | 0.068 | False |
| MDW | Change 1–3 | **0.003** | **0.022** | True |
| MDW | Change 1–7 | **0.012** | **0.045** | True |
| MDW | Day 3 | **0.003** | **0.022** | True |
| MDW | Day 7 | **0.007** | **0.035** | True |
| WBC | Change 3–7 | **0.033** | 0.068 | False |
| WBC | Day 3 | 0.805 | 0.821 | False |
| WBC | Day 7 | 0.070 | 0.096 | False |

Abbreviations: CRP: C-reactive protein (mg/L); MDW: monocyte distribution width; Q_BH: Benjamini–Hochberg adjusted q-value; WBC: white blood cells (× 10^9^/L).

Timepoint definitions: Day 0: preoperative day (before surgery); Day 1: postoperative day 1; Day 3: postoperative day 3; Day 7: postoperative day 7.

The change values represent differences between the specified days (e.g., Day 0−1 = Day 1 value minus Day 0 value).

Values are presented with three decimals. Statistical significance after Benjamini–Hochberg correction is indicated in the last column (q<0.05).

Notes:Statistically significant p values (< 0.05) are highlighted in bold for clarity.
