# Supplementary material for: Evaluation of monocyte distribution width as a predictive factor for early complications of pancreatic surgery (pancreaticoduodenectomy): a retrospective cohort study
Source: BMC Surg. 2025 Nov 3;25:518. doi: 10.1186/s12893-025-03272-2 (PMC12581569; doi:10.1186/s12893-025-03272-2)
Supplement: Supplementary file 2 — Supplementary Material 2 [file 12893_2025_3272_MOESM2_ESM.docx]

**Supplementary Table S2a. Machine learning performance for overall complications**

| Model | Accuracy | Sensitivity | Specificity | F1 | ROC-AUC | Precision |
| --- | --- | --- | --- | --- | --- | --- |
| Logistic Regression | 0.743 ± 0.084 | 0.760 ± 0.049 | 0.719 ± 0.161 | 0.785 ± 0.064 | 0.808 ± 0.076 | 0.816 ± 0.099 |
| Decision Tree | 0.732 ± 0.058 | 0.780 ± 0.117 | 0.657 ± 0.172 | 0.778 ± 0.050 | 0.719 ± 0.062 | 0.793 ± 0.078 |
| Random Forest | 0.818 ± 0.066 | 0.880 ± 0.075 | 0.719 ± 0.122 | 0.854 ± 0.053 | 0.820 ± 0.111 | 0.833 ± 0.058 |
| SVM (RBF) | 0.816 ± 0.104 | 0.860 ± 0.102 | 0.752 ± 0.187 | 0.852 ± 0.082 | 0.809 ± 0.128 | 0.852 ± 0.102 |
| k-NN | 0.779 ± 0.095 | 0.740 ± 0.102 | 0.843 ± 0.183 | 0.803 ± 0.079 | 0.824 ± 0.102 | 0.895 ± 0.110 |
| Naïve Bayes | 0.706 ± 0.150 | 0.700 ± 0.210 | 0.719 ± 0.237 | 0.729 ± 0.171 | 0.834 ± 0.087 | 0.803 ± 0.127 |
| LDA | 0.768 ± 0.084 | 0.760 ± 0.049 | 0.781 ± 0.167 | 0.802 ± 0.063 | 0.795 ± 0.084 | 0.853 ± 0.100 |
| QDA | 0.706 ± 0.065 | 0.840 ± 0.136 | 0.490 ± 0.180 | 0.774 ± 0.058 | 0.755 ± 0.078 | 0.728 ± 0.042 |

Legend: Values are presented as mean ± standard deviation. Abbreviations: F1: F1-score; k-NN:k-nearest neighbors; LDA: linear discriminant analysis; Precision: positive predictive value; QDA: quadratic discriminant analysis; ROC-AUC: area under the receiver operating characteristic curve; SVM (RBF): support vector machine with radial basis function kernel.

**Supplementary Table S2b. Machine learning performance for anastomotic leaks**

| Model | Accuracy | Sensitivity | Specificity | F1 | ROC-AUC | Precision |
| --- | --- | --- | --- | --- | --- | --- |
| Logistic Regression | 0.731 ± 0.092 | 0.250 ± 0.129 | 0.846 ± 0.109 | 0.267 ± 0.162 | 0.612 ± 0.127 | 0.357 ± 0.340 |
| Decision Tree | 0.721 ± 0.093 | 0.133 ± 0.163 | 0.864 ± 0.089 | 0.157 ± 0.204 | 0.499 ± 0.100 | 0.250 ± 0.387 |
| Random Forest | 0.770 ± 0.091 | 0.067 ± 0.133 | 0.938 ± 0.090 | 0.080 ± 0.160 | 0.620 ± 0.157 | 0.100 ± 0.200 |
| SVM (RBF) | 0.805 ± 0.021 | 0.000 ± 0.000 | 1.000 ± 0.000 | 0.000 ± 0.000 | 0.600 ± 0.163 | 0.000 ± 0.000 |
| k-NN | 0.805 ± 0.069 | 0.200 ± 0.163 | 0.954 ± 0.062 | 0.267 ± 0.226 | 0.562 ± 0.179 | 0.467 ± 0.452 |
| Naïve Bayes | 0.768 ± 0.078 | 0.367 ± 0.221 | 0.862 ± 0.102 | 0.361 ± 0.198 | 0.647 ± 0.174 | 0.433 ± 0.327 |
| LDA | 0.707 ± 0.124 | 0.200 ± 0.163 | 0.831 ± 0.149 | 0.202 ± 0.189 | 0.595 ± 0.133 | 0.283 ± 0.371 |
| QDA | 0.780 ± 0.051 | 0.000 ± 0.000 | 0.969 ± 0.062 | 0.000 ± 0.000 | 0.616 ± 0.146 | 0.000 ± 0.000 |

Legend: Values are presented as mean ± standard deviation. Abbreviations: F1: F1-score; k-NN:k-nearest neighbors; LDA: linear discriminant analysis; Precision: positive predictive value; QDA: quadratic discriminant analysis; ROC-AUC: area under the receiver operating characteristic curve; SVM (RBF): support vector machine with radial basis function kernel.

**Supplementary Table S2c. Machine learning performance for CR-POPF (grade B/C)**

| Model | Accuracy | Sensitivity | Specificity | F1 | ROC-AUC | Precision |
| --- | --- | --- | --- | --- | --- | --- |
| Logistic Regression | 0.805 ± 0.069 | 0.000 ± 0.000 | 0.917 ± 0.079 | 0.000 ± 0.000 | 0.621 ± 0.212 | 0.000 ± 0.000 |
| Decision Tree | 0.804 ± 0.062 | 0.100 ± 0.200 | 0.903 ± 0.072 | 0.080 ± 0.160 | 0.501 ± 0.097 | 0.067 ± 0.133 |
| Random Forest | 0.878 ± 0.004 | 0.000 ± 0.000 | 1.000 ± 0.000 | 0.000 ± 0.000 | 0.521 ± 0.200 | 0.000 ± 0.000 |
| SVM (RBF) | 0.878 ± 0.004 | 0.000 ± 0.000 | 1.000 ± 0.000 | 0.000 ± 0.000 | 0.335 ± 0.178 | 0.000 ± 0.000 |
| k-NN | 0.878 ± 0.004 | 0.000 ± 0.000 | 1.000 ± 0.000 | 0.000 ± 0.000 | 0.517 ± 0.136 | 0.000 ± 0.000 |
| Naïve Bayes | 0.817 ± 0.040 | 0.100 ± 0.200 | 0.916 ± 0.070 | 0.067 ± 0.133 | 0.531 ± 0.113 | 0.050 ± 0.100 |
| LDA | 0.817 ± 0.084 | 0.100 ± 0.200 | 0.917 ± 0.079 | 0.133 ± 0.267 | 0.600 ± 0.161 | 0.200 ± 0.400 |
| QDA | 0.878 ± 0.004 | 0.000 ± 0.000 | 1.000 ± 0.000 | 0.000 ± 0.000 | 0.500 ± 0.000 | 0.000 ± 0.000 |

Legend: Values are presented as mean ± standard deviation. Abbreviations: CR-POPF: clinically relevant postoperative pancreatic fistula; F1: F1-score; k-NN:k-nearest neighbors; LDA: linear discriminant analysis; Precision: positive predictive value; QDA: quadratic discriminant analysis; ROC-AUC: area under the receiver operating characteristic curve; SVM (RBF): support vector machine with radial basis function kernel.

**Supplementary Table S2d. Machine learning performance for surgical site infections**

| Model | Accuracy | Sensitivity | Specificity | F1 | ROC-AUC | Precision |
| --- | --- | --- | --- | --- | --- | --- |
| Logistic Regression | 0.647 ± 0.038 | 0.419 ± 0.137 | 0.796 ± 0.064 | 0.474 ± 0.123 | 0.640 ± 0.117 | 0.573 ± 0.065 |
| Decision Tree | 0.696 ± 0.071 | 0.633 ± 0.085 | 0.738 ± 0.099 | 0.626 ± 0.086 | 0.686 ± 0.071 | 0.626 ± 0.112 |
| Random Forest | 0.685 ± 0.084 | 0.514 ± 0.149 | 0.798 ± 0.140 | 0.560 ± 0.110 | 0.756 ± 0.101 | 0.687 ± 0.198 |
| SVM (RBF) | 0.610 ± 0.043 | 0.424 ± 0.105 | 0.733 ± 0.105 | 0.461 ± 0.066 | 0.689 ± 0.078 | 0.533 ± 0.081 |
| k-NN | 0.685 ± 0.062 | 0.333 ± 0.138 | 0.918 ± 0.076 | 0.445 ± 0.139 | 0.667 ± 0.131 | 0.793 ± 0.194 |
| Naïve Bayes | 0.660 ± 0.055 | 0.581 ± 0.164 | 0.713 ± 0.134 | 0.571 ± 0.077 | 0.745 ± 0.093 | 0.595 ± 0.093 |
| LDA | 0.646 ± 0.023 | 0.414 ± 0.153 | 0.796 ± 0.064 | 0.467 ± 0.131 | 0.637 ± 0.099 | 0.568 ± 0.063 |
| QDA | 0.523 ± 0.081 | 0.386 ± 0.242 | 0.607 ± 0.200 | 0.369 ± 0.145 | 0.510 ± 0.116 | 0.400 ± 0.122 |

Legend: Values are presented as mean ± standard deviation. Abbreviations: F1: F1-score; k-NN:k-nearest neighbors; LDA: linear discriminant analysis; Precision: positive predictive value; QDA: quadratic discriminant analysis; ROC-AUC: area under the receiver operating characteristic curve; SVM (RBF): support vector machine with radial basis function kernel.
