# Supplementary material for: Evaluation of monocyte distribution width as a predictive factor for early complications of pancreatic surgery (pancreaticoduodenectomy): a retrospective cohort study
Source: BMC Surg. 2025 Nov 3;25:518. doi: 10.1186/s12893-025-03272-2 (PMC12581569; doi:10.1186/s12893-025-03272-2)
Supplement: Supplementary file 3 — Supplementary Material 3 [file 12893_2025_3272_MOESM3_ESM.docx]

**Supplementary Table 3a. Univariate analyses of factors associated with surgical site infection**

| Variable | SSI Absent, n (%) | SSI Present, n (%) | p value |
| --- | --- | --- | --- |
| Age (years) | 61 (27–84) | 66 (46–81) | 0.236^a^ |
| Sex |  |  | 0.170^b^ |
| Female | 23 (46.9) | 10 (30.3) |  |
| Male | 26 (53.1) | 23 (69.7) |  |
| Smoking |  |  | 0.658^c^ |
| Absent | 25 (51.0) | 15 (45.5) |  |
| Present | 24 (49.0) | 18 (54.5) |  |
| Comorbidity |  |  | 0.634^c^ |
| Absent | 16 (32.7) | 9 (27.3) |  |
| Present | 33 (67.3) | 24 (72.7) |  |
| Clavien–Dindo |  |  | **0.004**^b^ |
| 1–2 | 38 (77.6) | 15 (45.5) |  |
| 3–4 | 11 (22.4) | 18 (54.5) |  |
| Early mortality |  |  | 0.999^c^ |
| Absent | 47 (95.9) | 32 (97.0) |  |
| Present | 2 (4.1) | 1 (3.0) |  |
| Pancreas texture |  |  | 0.191^d^ |
| Soft | 19 (38.8) | 8 (24.2) |  |
| Intermediate | 24 (49.0) | 23 (69.7) |  |
| Hard | 6 (12.2) | 2 (6.1) |  |
| Preoperative biliary drainage |  |  | **0.001**^d^ |
| Absent | 28 (57.1) | 5 (15.2) |  |
| ERCP stent | 11 (22.4) | 18 (54.5) |  |
| PBD | 10 (20.4) | 10 (30.3) |  |
| Number of RBC transfusions* | 1 (0–9) | 1 (0–7) | 0.089^a^ |
| Number of FFP transfusions* | 0 (0–23) | 2 (0–11) | **0.029**^a^ |
| Pre-op albumin (g/dL) | 3.7 (2.8–5.1) | 3.4 (1.8–4.3) | **0.046**^a^ |
| Intraoperative blood loss (mL) | 200 (50–650) | 250 (150–600) | 0.120^a^ |
| Pancreatic duct diameter (mm) | 3 (2–15) | 4 (2–9) | 0.094^a^ |
| Mortality |  |  | 0.455^c^ |
| Alive | 37 (75.5) | 22 (66.7) |  |
| Exitus | 12 (24.5) | 11 (33.3) |  |
| Length of hospital stay (days) | 12 (7–53) | 19 (9–43) | **<0.001**^a^ |
| Follow-up (months) | 11 (1–35) | 9 (1–34) | 0.630^a^ |

Abbreviations: ERCP: endoscopic retrograde cholangiopancreatography; FFP: fresh frozen plasma; PBD: percutaneous biliary drainage; RBC: red blood cell.

Notes:

^a^ Mann‒Whitney U test.

^b^ Chi-square test.

^c^ Fisher’s exact test.

^d^ Friedman fisher test.

Statistically significant p values (< 0.05) are highlighted in bold for clarity.

Univariate analysis of demographic, perioperative, and clinical variables in relation to SSI. Continuous data are presented as median (range). Categorical data are shown as n (%). p-values represent statistical comparisons between SSI-present and SSI-absent groups

**Supplementary Table 3b. ROC analyses of significant parameters for surgical site infection**

| Variable | Timepoint | Cut-off | Sensitivity (%) | Specificity (%) | +PV (%) | −PV (%) | +LR | −LR | AUC ± SE | p value |
| --- | --- | --- | --- | --- | --- | --- | --- | --- | --- | --- |
| MDW | Day 3 | >23.4 | 78.8 | 61.2 | 56.5 | 80.6 | 2.0 | 0.3 | 0.699 ± 0.058 | **0.002** |
| MDW | Day 7 | >23.2 | 69.7 | 71.4 | 62.2 | 77.8 | 2.4 | 0.4 | 0.750 ± 0.054 | **<0.001** |
| CRP | Day 0 | >13.1 | 69.0 | 67.0 | 59.0 | 76.0 | 2.1 | 0.4 | 0.696 ± 0.059 | **0.003** |
| CRP | Day 3 | >255 | 45.0 | 77.0 | 57.0 | 67.0 | 2.0 | 0.7 | 0.651 ± 0.062 | **0.021** |
| CRP | Day 7 | >92.5 | 67.0 | 65.0 | 56.0 | 74.0 | 2.0 | 0.5 | 0.714 ± 0.056 | **0.001** |
| WBC | Day 7 | >11.9 | 48.0 | 77.6 | 59.0 | 69.0 | 2.0 | 0.6 | 0.660 ± 0.063 | **0.015** |
| CRP change | Day 0–7 | >66.4 | 60.0 | 61.0 | 51.0 | 69.0 | 1.56 | 0.6 | 0.634 ± 0.064 | **0.040** |
| CRP change | Day 1–7 | >36 | 72.0 | 63.0 | 57.0 | 77.0 | 2.0 | 0.4 | 0.674 ± 0.061 | **0.008** |

Abbreviations: AUC: area under the curve; CRP: C-reactive protein; +LR: positive likelihood ratio; −LR: negative likelihood ratio; MDW: monocyte distribution width; +PV: positive predictive value; −PV: negative predictive value; ROC: receiver operating characteristic; SE: standard error; SSI: surgical site infection; WBC: white blood cell

Reference standard: SSI presence.

Timepoint definitions: Day 0: preoperative day (before surgery); Day 1: postoperative day 1; Day 3: postoperative day 3; Day 7: postoperative day 7.

The change values represent differences between the specified days (e.g., Day 0−1 = Day 1 value minus Day 0 value).

**Supplementary Table 3c. Multivariate logistic regression analysis of factors associated with surgical site infection**

| Variable | B (SE) | p value | Odds Ratio (95% CI) |
| --- | --- | --- | --- |
| Age | 0.02 (0.02) | 0.417 | 1.019 [0.974–1.066] |
| Sex (Male) | 1.14 (0.60) | 0.058 | 3.123 [0.963–10.129] |
| Preoperative albumin (↓) | 1.71 (0.55) | **0.002** | 5.528 [1.884–16.218] |
| FFP transfusion units* (↑) | 0.25 (0.10) | **0.013** | 1.289 [1.056–1.573] |
| FFP transfusion status* (Yes) | 1.75 (0.71) | **0.013** | 5.775 [1.445–23.088] |
| MDW Day 7 (↑) | 0.22 (0.09) | **0.012** | 1.244 [1.049–1.475] |
| CRP Day 7 (↑) | 0.01 (0.00) | **0.030** | 1.01 [1.001–1.019] |

Abbreviations: B: regression coefficient; CI: confidence interval; CRP: C-reactive protein; FFP: fresh frozen plasma; MDW: monocyte distribution width; SE: standard error.

Classification accuracy: SSI absent: 89.8%; SSI present: 60.6%; Overall: 78.0%.

Timepoint definitions: Day 0: preoperative day (before surgery); Day 1: postoperative day 1; Day 3: postoperative day 3; Day 7: postoperative day 7.

Notes: Multivariate logistic regression analysis using backward stepwise Wald method. Model adjusted for age and sex.

* Represents transfusion status and units within operation and the first 7 postoperative days.

The arrows indicate the direction of association:

(↑) = Increased value associated with higher risk.

(↓) = Decreased value associated with higher risk.
