# Supplementary material for: Evaluation of monocyte distribution width as a predictive factor for early complications of pancreatic surgery (pancreaticoduodenectomy): a retrospective cohort study
Source: BMC Surg. 2025 Nov 3;25:518. doi: 10.1186/s12893-025-03272-2 (PMC12581569; doi:10.1186/s12893-025-03272-2)
Supplement: Supplementary file 4 — Supplementary Material 4 [file 12893_2025_3272_MOESM4_ESM.docx]

**Supplementary Figure 1:** **Kaplan–Meier Curves of Overall Survival by Complication Status**


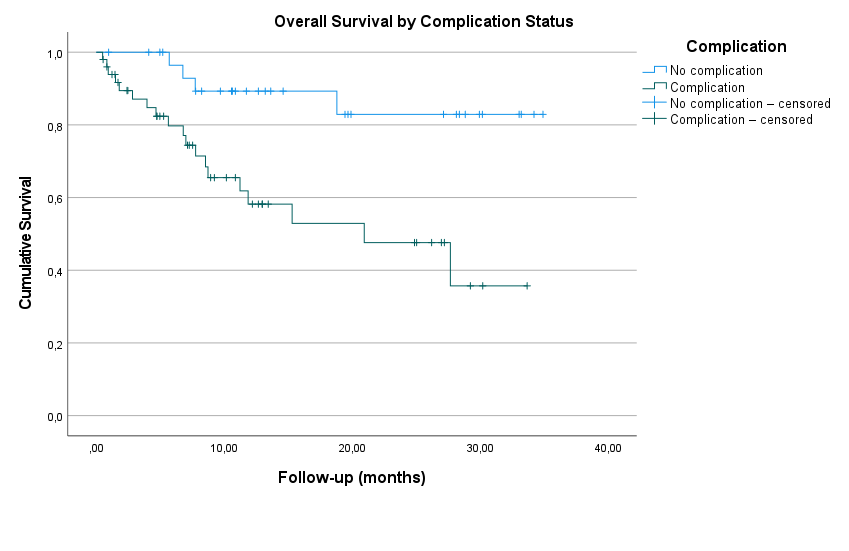


**Notes:** Patients with complications (green line) showed significantly poorer overall survival compared with those without complications (blue line). Log-rank test, p = 0.003.
